# Changes in Erectile Function, Genital Self-Image, and Psychological Distress in Men After Bariatric Surgery: A Single-Centre Prospective Cohort Study

**DOI:** 10.3390/jcm15166276

**Published:** 2026-08-13

**Authors:** Magdalena Sternau, Mateusz Czajkowski, Monika Proczko-Stepaniak, Marcin Matuszewski

**Affiliations:** 1 Department of Urology, Medical University of Gdańsk, 80-210 Gdańsk, Poland; mateusz.czajkowski@gumed.edu.pl (M.C.); marcin.matuszewski@gumed.edu.pl (M.M.); 2 Department of General, Endocrine and Transplant Surgery, Medical University of Gdańsk, 80-210 Gdańsk, Poland; mproczko@gumed.edu.pl

**Keywords:** bariatric surgery, obesity, erectile dysfunction, male sexual function, genital self-image, psychological distress, sexual behaviour, IIEF-5, MGSIS-7

## Abstract

**Background**: Severe obesity is a chronic condition that adversely affects physical health and quality of life, including sexual function. However, its impact on multidimensional psychosexual outcomes remains unexplored. This study aimed to assess changes in erectile function, genital self-image, and psychological distress in men who underwent bariatric surgery. **Methods**: This prospective cohort study involved 50 male participants undergoing primary bariatric surgery who were evaluated within 30 days prior to the surgical procedure and at a minimum of 12 months postoperatively. Standardised questionnaires were used, including the International Index of Erectile Function-5 (IIEF-5), Male Genital Self-Image Scale (MGSIS-7), and Hospital Anxiety and Depression Scale–Modified (HADS-M). Secondary outcomes encompassed parameters of weight loss, sexual activity, and selected aspects of sexual behaviour, such as the utilisation of various sexual positions, their associated pleasure, and frequency. **Results**: Significant postoperative improvements were observed in BMI reduction from 42.8 kg/m^2^ to 31.7 kg/m^2^ (*p* < 0.001), erectile function (IIEF-5 improved from 16.3 to 19.6; *p* < 0.001), genital self-image (MGSIS-7 improved from 19.1 to 21.9; *p* < 0.001), and psychological distress (HADS-M reduction from 13.3 to 8.7; *p* < 0.001). Glycated haemoglobin decreased significantly (5.89% to 5.31%; *p* < 0.001), obstructive sleep apnoea severity improved (*p* < 0.001), and remission or clinically meaningful improvement of at least one major metabolic comorbidity was documented in 24 of 37 participants (64.9%). Sexual activity increased from 37/50 (74.0%) to 43/50 (86.0%), although this change was not statistically significant (*p* = 0.114). After FDR correction, frequency increased for 3 of the assessed positions, whereas pleasure increased only for one of them. Weight loss magnitude was not significantly correlated with changes in the IIEF-5 or MGSIS-7 scores. A greater reduction in the HADS-M score correlated with a greater improvement in the IIEF-5 score (ρ = −0.307, *p* = 0.030). **Conclusions**: Bariatric surgery was associated with improvements in erectile function, genital self-image, and psychological distress, alongside marked glycaemic and sleep-related recovery. Because the psychosexual changes were not statistically associated with the magnitude of weight loss and of metabolic and respiratory improvement, and because the design was uncontrolled, these exploratory findings indicate association rather than causation and support the incorporation of psychosexual and mental health assessment into postoperative bariatric care.

## 1. Introduction

Severe obesity is a chronic multisystem disease associated with cardiometabolic morbidity and impaired health-related quality of life, including sexual well-being. In men with obesity, sexual function is an important yet frequently under-recognised dimension of quality of life. Metabolic and bariatric surgery is now established as the most effective treatment for durable weight reduction in severe obesity, and its benefits appear to extend beyond body weight and remission of comorbidities to broader psychosocial and sexual health outcomes [1,2,3,4]. The relationship between obesity and male sexual dysfunction is complex, both biologically and psychosocially. Proposed mechanisms include androgen deficiency related to increased aromatase activity, endothelial dysfunction, chronic low-grade inflammation, insulin resistance, and obesity-related comorbidities, alongside poorer body image, lower self-esteem, anxiety, and depressive symptoms, all of which may adversely affect erectile function and sexual satisfaction [1,2,3,5,6]. The metabolic benefits of bariatric surgery are well-documented, including enhanced glycemic control, diabetes remission, and a decrease in diabetes medication usage. Furthermore, bariatric surgery contributes to improved blood pressure and lipoprotein profiles, which are recognised risk factors for sexual dysfunction in men [7,8,9,10]. A comprehensive study involving nearly 16,000 participants demonstrated that individuals experiencing dissatisfaction with their body weight exhibit a higher prevalence of depression, irrespective of their Body Mass Index (BMI) [11]. A separate study demonstrated the impact of negative self-perception regarding penile appearance on erectile dysfunction [12].

Available studies using IIEF-based measures after bariatric surgery generally report postoperative improvement, although the effects vary across domains, procedures, and follow-up intervals. A 2023 systematic review and meta-analysis including 14 longitudinal studies and 508 patients demonstrated significant improvements in overall IIEF, erectile function, sexual desire, intercourse satisfaction, and overall satisfaction, whereas orgasmic function did not improve significantly [4]. A more recent systematic review and meta-analysis including 21 studies and 695 participants likewise reported postoperative improvement in IIEF-5 and IIEF-15 scores and an increase in total testosterone levels, while emphasising heterogeneity in study design, surgical procedures, follow-up duration, and sample size [13]. Individual prospective studies have similarly reported improvements after bariatric surgery [5,6,14,15], although postoperative recovery may be nonlinear and domain-specific [16,17].

Taken together, current evidence supports the beneficial effect of bariatric surgery on male sexual function; however, most previous studies have focused on IIEF outcomes, hormonal measures, health-related quality of life, or broader sexual satisfaction rather than on a multidimensional psychosexual construct. In particular, prospective studies simultaneously evaluating erectile function, genital self-image, and psychological distress in the same cohort of men remain scarce. It also remains unclear whether postoperative sexual improvement is explained primarily by the magnitude of weight loss or whether it is more closely related to changes in body-related sexual self-perception and psychological well-being [2,3,4,15,16,17,18].

Based on the available evidence, we hypothesised that postoperative assessments would show higher IIEF-5 and MGSIS-7 scores and lower HADS-M scores than preoperative assessments, and that improvement in erectile function would be more closely associated with reduced psychological distress than with the magnitude of weight loss. Accordingly, the primary objective of this prospective cohort study was to assess within-participant changes in erectile function, genital self-image, and psychological distress after bariatric surgery using the IIEF-5, MGSIS-7, and HADS-M, respectively. The secondary objectives were to assess changes in sexual activity and sexual behaviour and to explore whether psychosexual changes were associated with the magnitude of weight loss, baseline cardiometabolic comorbidity, obstructive sleep apnoea and changes in psychological distress.

## 2. Materials and Methods

### 2.1. Study Design and Setting

This single-centre prospective cohort study was conducted at the Medical University of Gdańsk (Department of General, Endocrine, and Transplant Surgery and Department of Urology).

### 2.2. Participants and Recruitment

Men were recruited between 2023 and 2024 and reassessed at least 1 year after bariatric surgery.

The patients completed questionnaires 30 days before the surgery and at least 1 year after. The completion was self-reported, without the influence of the researchers. The inclusion criteria were age ≥18 years, eligibility for bariatric surgery, and written informed consent. The exclusion criteria were pharmacological treatment for erectile dysfunction, previously diagnosed and/or pharmacologically treated depression or anxiety, testosterone replacement therapy, extensive reconstructive surgery in the genital area, and previous bariatric surgery. Because pharmacological treatment for erectile dysfunction and previously diagnosed or pharmacologically treated depression or anxiety were exclusion criteria, no participant was receiving a phosphodiesterase type 5 inhibitor, testosterone replacement, an antidepressant, or an anxiolytic at enrolment. Remaining chronic pharmacotherapy was recorded as free text at both time points and was summarised as the number of chronic agents per patient; the free-text entries were not reclassified into pharmacological classes with initiation, discontinuation, and dose-modification status, since dose information was inconsistently documented.

### 2.3. Surgical Procedures and Postoperative Follow-Up

**Figure 1:**
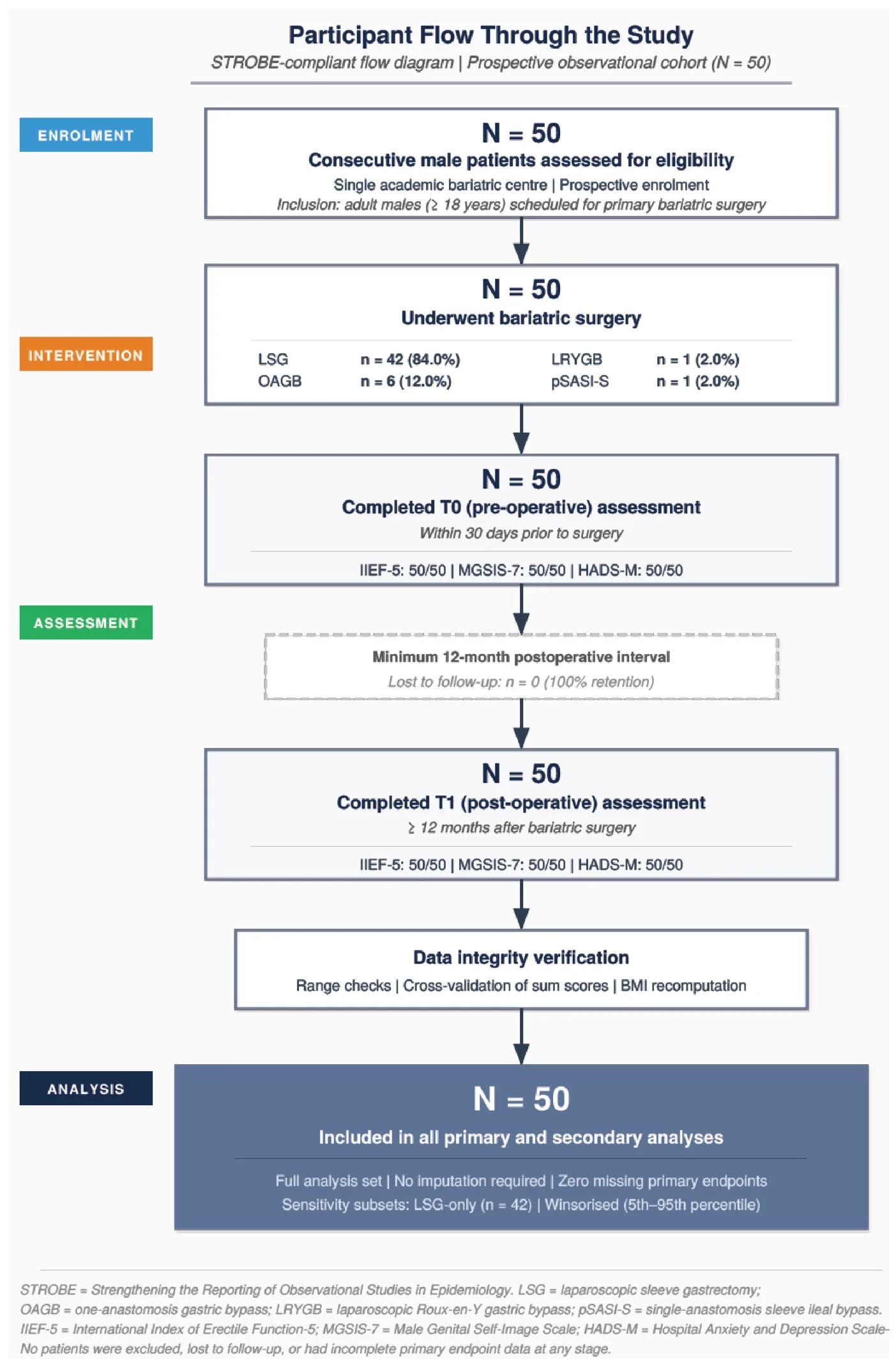
Participant flow through the study. Consecutive adult men eligible for primary bariatric surgery were enrolled, assessed preoperatively within 30 days before surgery, and reassessed at least 12 months postoperatively. Complete paired data were available for all the primary outcomes.

The surgical procedures included laparoscopic sleeve gastrectomy in 42 patients (84.0%), one-anastomosis gastric bypass in six (12.0%), laparoscopic Roux-en-Y gastric bypass in one (2.0%), and single-anastomosis sleeve ileal bypass in one (2.0%). Procedure selection was based on clinical indications and surgeon judgement, which may have introduced selection bias. The interval between surgery and the follow-up assessment was recorded individually for every participant. The bariatric unit managed perioperative complications, reoperations, readmissions, and nutritional supplementation under a uniform institutional protocol and were not transcribed into the urological study database; they are therefore not reported. Because body mass was measured at two time points only, weight regain relative to a postoperative nadir could not be derived. No participant was lost to follow-up, and complete paired data were available for all the primary outcomes (Figure 1).

### 2.4. Outcomes and Measurement Instruments

**Figure 2:**
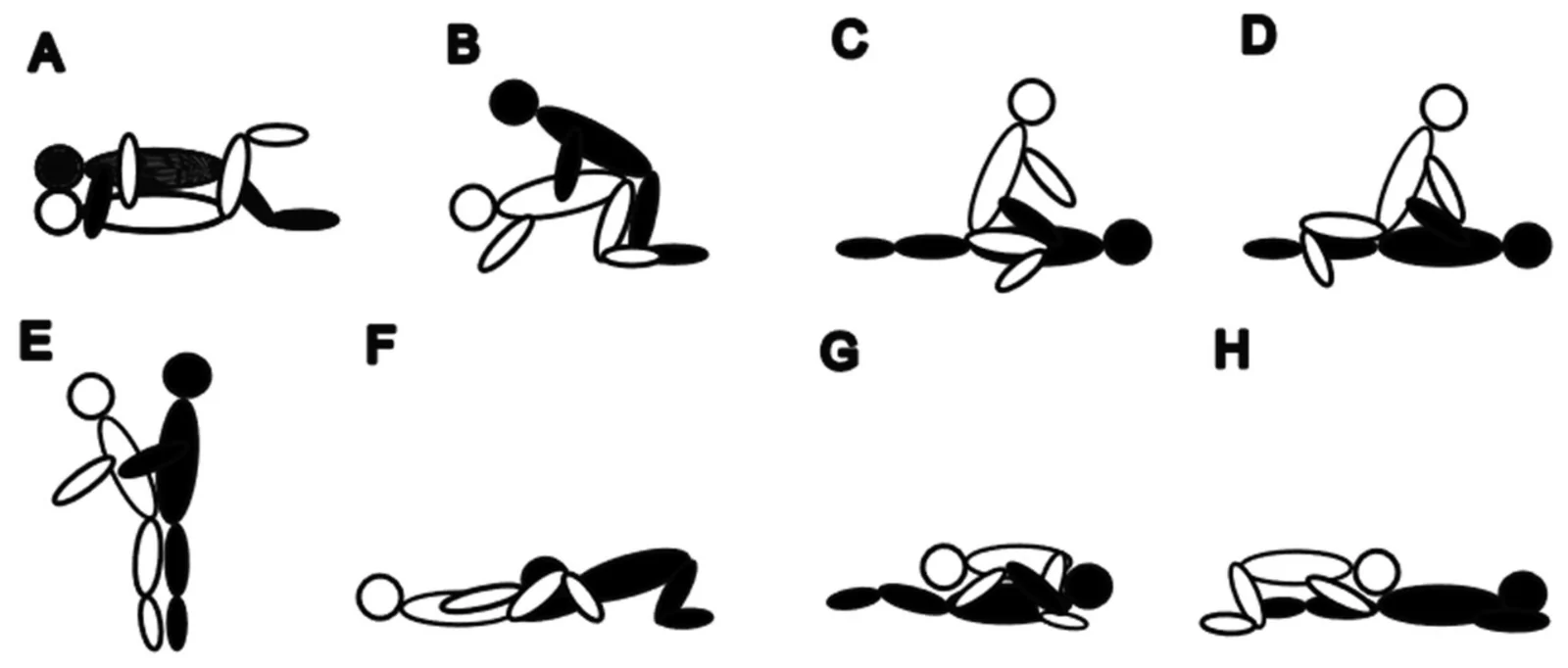
Schematic representation of the eight predefined partnered sexual positions (**A**–**H**) assessed using the study-specific questionnaire. The dark silhouette denotes the study participants, and the light silhouette denotes the partner. The item set was developed for this study and was not independently validated; therefore, position-related findings should be interpreted as exploratory.

The primary outcomes focused on within-subject changes in erectile function, genital self-image, and psychological distress levels. These were assessed using the International Index of Erectile Function-5 (IIEF-5), where scores of 5–7 indicated severe erectile dysfunction, 8–11 moderate, 12–16 mild to moderate, 17–21 mild, and 22–25 no erectile dysfunction. Men who scored less than 5 points were not sexually active [19]. Genital self-image was assessed using the Male Genital Self-Image Scale (MGSIS-7), a seven-item instrument scored on a 4-point Likert scale, with total scores ranging from 7 to 28, with higher scores indicating more favourable genital self-image. A score of ≥23 was used to indicate satisfied genital self-image [20,21]. Psychological distress was assessed using the Hospital Anxiety and Depression Scale–Modified (HADS-M). Scores were interpreted as indicators of symptom burden rather than formal psychiatric diagnosis. For anxiety and depressive symptom subscales, scores of 0–7 were interpreted as normal, 8–10 as borderline, and ≥11 as elevated symptom burden, in line with the commonly used HADS interpretative thresholds. The HADS-M total score was analysed as a continuous measure of overall psychological distress [22,23]. Secondary outcomes included changes in body weight and body mass index (BMI), glycated haemoglobin (HbA1c), obstructive sleep apnoea (OSA) status and severity grade, diagnosed using polysomnography (the severity was assessed according to apnoea–hypopnea index (AHI), with 5 to <15 being mild OSA, 15 to 30 moderate and >30 severe [24]), the number of chronic medications, sexual activity status, monthly coital frequency, frequency and pleasure related to eight predefined sexual positions (Figure 2), and clinician-adjudicated remission or improvement of the major metabolic comorbidities. Baseline hypertension, type 2 diabetes mellitus, and hyperlipidaemia were evaluated as potential effect modifiers. The percentage total body weight loss (%TBWL) was calculated as ((weight at T0 − weight at T1)/weight at T0) × 100. Blood pressure, fasting glucose, fasting insulin, lipid profile, sex hormones, and inflammatory markers were not part of the study protocol and were not collected. This study was reported in accordance with the Strengthening the Reporting of Observational Studies in Epidemiology (STROBE) guidelines for cohort studies.

### 2.5. Statistical Methods

Distributional assumptions were assessed using the Shapiro–Wilk test. Because the change scores were non-normally distributed and several outcomes were ordinal, the primary inferential framework was non-parametric. Pre–post comparisons were analysed using the Wilcoxon signed-rank test, with effect sizes expressed as rank-biserial correlations and 95% bias-corrected and accelerated bootstrap confidence intervals. Paired categorical variables were analysed using McNemar’s exact test. Spearman’s rank correlations were used to evaluate the associations between weight loss indices and changes in psychometric outcomes, as well as between changes in psychological distress and sexual outcomes. Change scores were compared between the comorbidity subgroups using the Mann–Whitney U test. Given the modest sample size, all analyses of associations and subgroup differences were interpreted as exploratory and hypothesis generating. All tests were two sided. A Bonferroni-adjusted threshold of *p* < 0.017 was applied to the three primary outcomes, whereas secondary and exploratory analyses used *p* < 0.05. False discovery rate correction using the Benjamini–Hochberg procedure was applied to the sexual position analyses. Analyses were performed using R version 4.2.1 (R Foundation for Statistical Computing, Vienna, Austria). Quantitative psychometric and anthropometric variables were analysed as continuous measures. Change scores (Δ = follow-up − baseline) were calculated for all primary outcomes and anthropometric indices. For descriptive analyses, IIEF-5 and HADS-M scores were additionally classified according to established clinical categories, whereas sexual activity was treated as a binary variable and measures of coital frequency and sexual-position use were analysed as ordinal variables. Sensitivity analyses were performed to evaluate the robustness of the findings. Primary outcome analyses were repeated in the laparoscopic sleeve gastrectomy subgroup, using parametric paired *t*-tests as an alternative analytical approach, and after winsorisation of continuous change scores at the 5th and 95th percentiles.

Additional analyses introduced during revision were post hoc and exploratory. Additional paired comparisons were analysed using the Wilcoxon signed-rank test with rank-biserial correlation effect sizes and bias-corrected and accelerated (BCa) bootstrap 95% confidence intervals (B = 10,000), whereas paired proportions were analysed using McNemar’s exact test. Associations between clinical variables and change scores were evaluated using Spearman’s rank correlation with percentile bootstrap 95% confidence intervals (B = 5000) and the Mann–Whitney U test, as appropriate. Changes in obstructive sleep apnoea severity across baseline severity strata were additionally assessed using the Cochran–Armitage trend test and Fisher’s exact test. The previously specified Bonferroni correction for the three primary outcomes remained unchanged, whereas Benjamini–Hochberg false discovery rate correction was applied within families of exploratory secondary analyses. Exploratory multivariable ordinary least squares regression models for changes in IIEF-5 and MGSIS-7 were adjusted for percentage total body weight loss, changes in HADS-M and HbA1c, postoperative improvement in OSA severity, follow-up duration, and age. Given the ratio of approximately eight observations per predictor, these models should be regarded as exploratory and hypothesis-generating. All additional analyses were conducted as complete-case analyses in R version 4.6.1 (R Foundation for Statistical Computing, Vienna, Austria) using a fixed random seed.

No formal a priori sample size calculation was performed. The sample size was determined by the number of eligible men who underwent primary bariatric surgery during the recruitment period and completed paired follow-up. The observational cohort was not prospectively registered in a clinical trial.

### 2.6. Ethics

The study protocol was approved by the Bioethics Committee for Scientific Research at the Medical University of Gdańsk on 9 December 2022 (NKBBN/792/2022). This study was conducted in accordance with the Declaration of Helsinki. Written informed consent was obtained from all participants prior to enrollment in the study.

## 3. Results

This study enrolled 50 consecutive adult men who underwent primary bariatric surgery, and none of them was lost in the follow-up. The cohort had a mean age of 44.2 ± 9.5 years and a baseline BMI of 42.8 ± 7.6 kg/m^2^. At baseline, 37 of 50 participants (74.0%) were sexually active, 35 (70.0%) had hypertension, 16 (32.0%) had type 2 diabetes mellitus, 14 (28.0%) had hyperlipidaemia, and 48 (96.0%) had obstructive sleep apnoea (full demographic data are presented in Table 1). Baseline mean scores indicated impairment across all three primary domains, with mean IIEF-5, MGSIS-7, and HADS-M scores of 16.3 ± 8.8, 19.1 ± 4.1, and 13.3 ± 6.8, respectively, indicating impairment. Following surgery, mean body weight decreased from 138.8 ± 21.4 kg to 102.7 ± 19.7 kg, and mean BMI decreased from 42.8 ± 7.6 kg/m^2^ to 31.7 ± 6.8 kg/m^2^ (both *p* < 0.001). Mean %TBWL was 25.8% ± 9.3%.

The mean interval between surgery and reassessment was 12.4 ± 0.6 months (median 12.0, IQR 12.0–13.0; range 12–14), with 30 participants (60.0%) assessed at 12 months, 18 (36.0%) at 13 months, and 2 (4.0%) at 14 months. All 50 participants attended the scheduled follow-up assessment. Follow-up duration was not associated with change in the IIEF-5 (ρ = −0.038, *p* = 0.794), MGSIS-7 (ρ = −0.099, *p* = 0.493), or HADS-M (ρ = −0.099, *p* = 0.493) scores.

**Figure 3:**
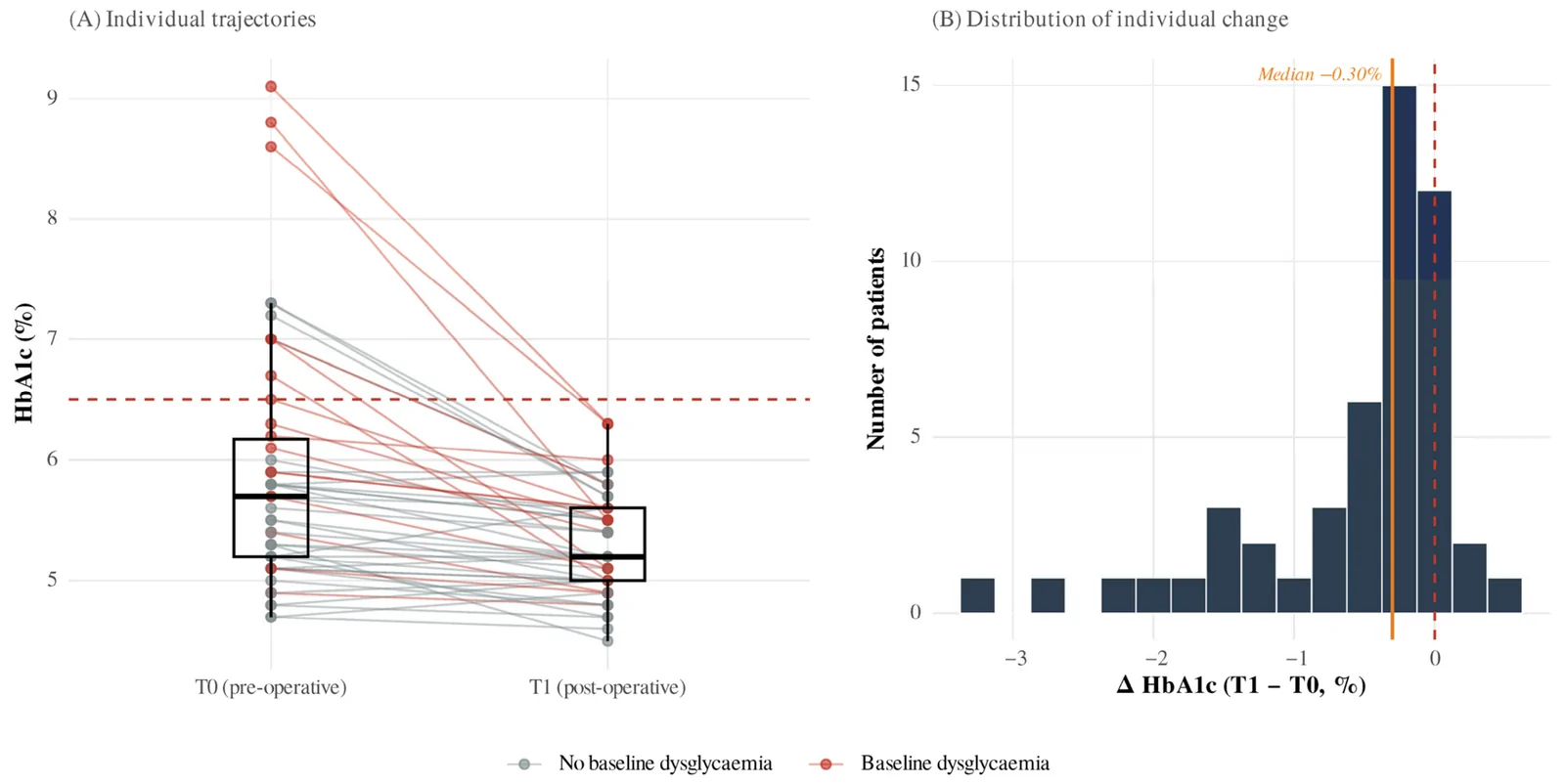
Glycated haemoglobin before and after bariatric surgery. Panel (**A**) shows individual trajectories between the preoperative and follow-up assessments, with the box plots denoting the median and interquartile range and the dashed line marking the 6.5% diabetes threshold; participants with type 2 diabetes mellitus or impaired fasting glucose at baseline (n = 16) are distinguished by colour. Panel (**B**) shows the distribution of individual change, with the vertical line at the cohort median. Wilcoxon signed-rank test: V = 80, *p* < 0.001; rank-biserial r = −0.864 (95% CI [−0.957, −0.655]). Data were complete for all 50 participants.

Metabolic and sleep-related parameters improved markedly (Table 2). Glycated haemoglobin decreased from 5.89 ± 1.03% to 5.31 ± 0.43% (mean change −0.57 ± 0.78%; V = 80, *p* < 0.001; r = −0.864, 95% CI [−0.957, −0.655]; Figure 3), and the reduction was proportionally greater among the 16 participants with baseline dysglycaemia (6.58 ± 1.27% to 5.46 ± 0.48%). Whereas 11 participants (22.0%) had an HbA1c of at least 6.5% before surgery, none did at follow-up (McNemar *p* = 0.003). Among the 37 participants with hypertension, type 2 diabetes mellitus, or hyperlipidaemia at baseline, remission or clinically meaningful improvement of at least one condition was documented in 24 (64.9%). The number of chronic medications declined from 3.9 ± 3.3 to 2.1 ± 2.3 per patient (*p* < 0.001).

**Figure 4:**
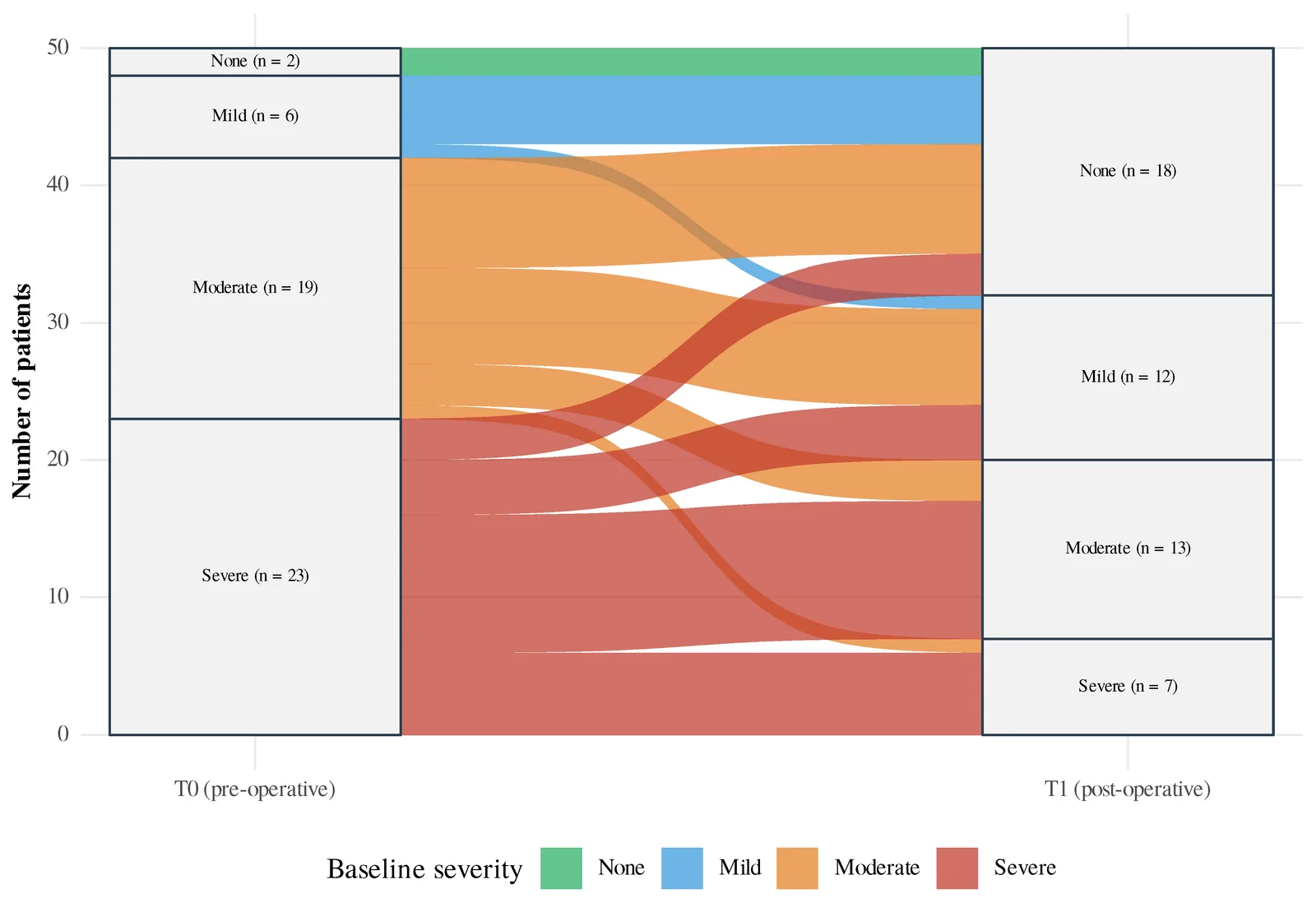
Transitions in obstructive sleep apnoea severity after bariatric surgery. Ribbon width is proportional to patient count and colour denotes the baseline severity grade; the number of participants in each severity category is given within the corresponding stratum at both time points. Wilcoxon signed-rank test for the ordinal severity grade: V = 12, *p* < 0.001; rank-biserial r = −0.968 (95% CI [−1.000, −0.825]). Complete resolution occurred in 16 of the 48 participants with baseline apnoea (33.3%) and improvement of at least one grade in 37 (77.1%).

Obstructive sleep apnoea was present in 48 participants (96.0%) before surgery, graded as mild in 6, moderate in 19, and severe in 23. At follow-up, the severity distribution had shifted towards milder disease (none 18, mild 12, moderate 13, severe 7), leaving apnoea in 32 participants (64.0%; McNemar *p* < 0.001). On the ordinal severity grade, the score decreased from 2.26 ± 0.83 to 1.18 ± 1.08 (V = 12, *p* < 0.001; r = −0.968, 95% CI [−1.000, −0.825]; Figure 4). Among the 48 participants with baseline apnoea, the index normalised in 16 (33.3%) and improved by at least one severity grade in 37 (77.1%). Normalisation depended strongly on baseline severity, occurring in 83.3% of those with mild, 42.1% of those with moderate, and 13.0% of those with severe apnoea (Cochran–Armitage test for trend, *p* < 0.001), whereas improvement by at least one grade was near-uniform across strata (83.3%, 78.9%, and 73.9%; *p* = 0.584). Neither baseline severity nor postoperative improvement was associated with change in the IIEF-5, MGSIS-7, or HADS-M scores.

Erectile function significantly improved after bariatric surgery. The mean IIEF-5 score increased from 16.3 ± 8.8 at T0 to 19.6 ± 6.8 at T1, corresponding to a mean change of 3.2 ± 6.4 points (Wilcoxon V = 640.5, *p* < 0.001; rank-biserial r = 0.729, 95% CI [0.392, 0.911]). The proportion of men classified as having no erectile dysfunction increased from 40.0% to 54.0%, and the moderate erectile dysfunction category was no longer present at follow-up. Genital self-image also improved significantly, with the mean MGSIS-7 score increasing from 19.1 ± 4.1 to 21.9 ± 4.1 (mean change 2.8 ± 3.2; V = 696.5, *p* < 0.001; r = 0.880, 95% CI [0.680, 0.965]). All seven MGSIS-7 items improved significantly, as shown in Table 3. Psychological distress declined significantly, with the mean HADS-M decreasing from 13.3 ± 6.8 to 8.7 ± 6.8 (mean change −4.6 ± 7.0; V = 115.5, *p* < 0.001; r = −0.767, 95% CI [−0.929, −0.447]). This reduction was primarily driven by improvements in anxiety and depressive symptoms (both *p* < 0.001), whereas hostility/irritability showed only a nominal decrease (*p* = 0.047) (Table 4).

**Figure 5:**
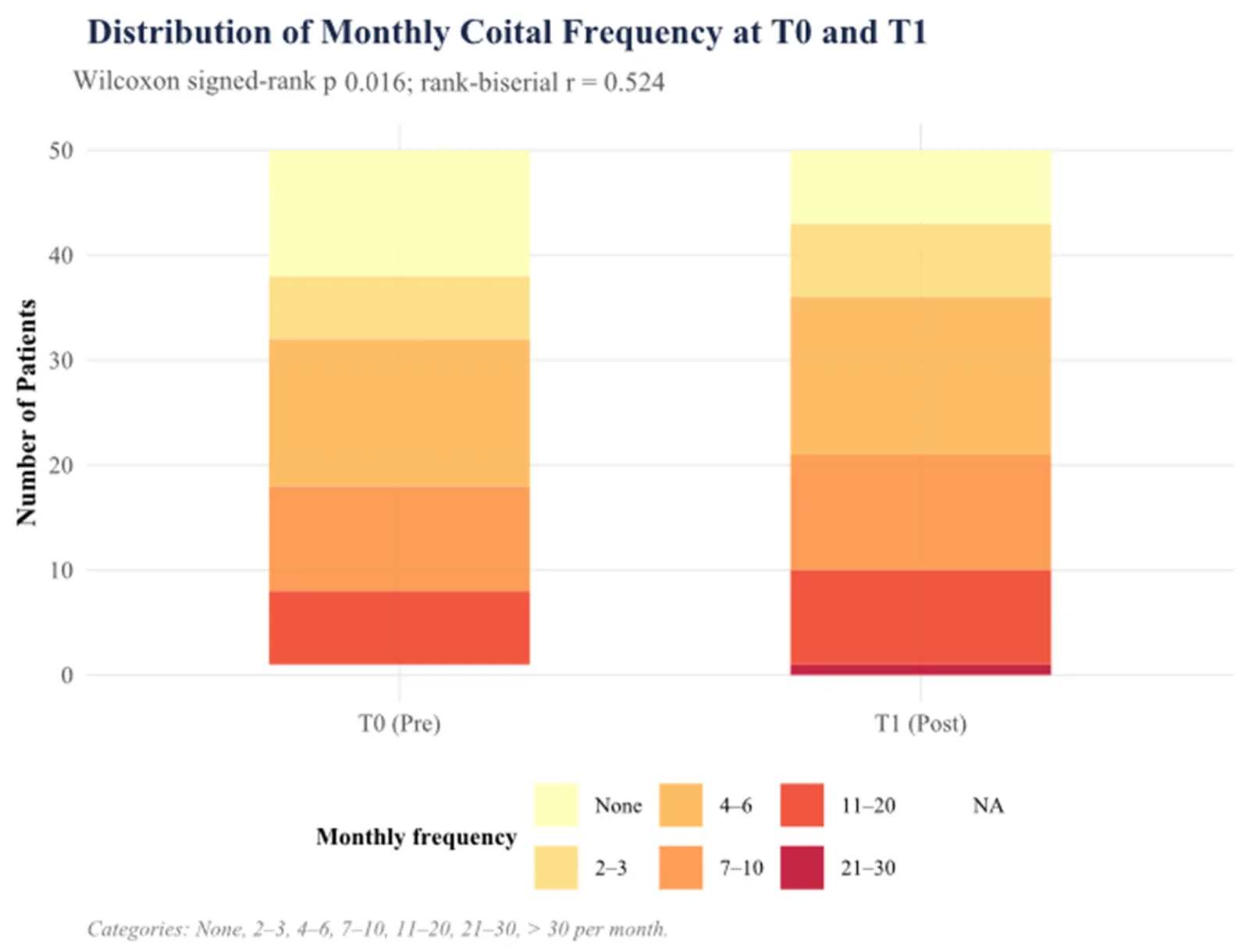
Distribution of monthly coital frequency at baseline and postoperative follow-up. The categories were presented as patient counts on a seven-level ordinal scale. Paired comparisons were performed using the Wilcoxon signed-rank test.

Exploratory sexual behaviour outcomes suggested postoperative changes. The proportion of sexually active men increased from 74.0% (n = 37) at baseline to 86.0% (n = 43) at follow-up. However, this change did not reach statistical significance in McNemar’s test (*p* = 0.114). Patients who were not sexually active did not complete positions questionnaire. The monthly coital frequency increased significantly (*p* = 0.016; r = 0.524; Figure 5). Relationship status was stable across the observation window: 44 participants (88.0%) were partnered at baseline and 43 (86.0%) at follow-up, with the status unchanged in 49 of 50 participants. Partnered men were more likely to be sexually active at both time points (Fisher exact test, *p* = 0.003 at T0 and *p* = 0.005 at T1) and reported higher monthly coital-frequency categories (Mann–Whitney U test, *p* = 0.019 at T0 and *p* < 0.001 at T1), but baseline relationship status was not associated with change in any primary outcome. Smoking, alcohol consumption, physical activity, and dietary change were not recorded with validated instruments and are not reported. Across the eight predefined sexual positions, all the mean changes in frequency and pleasure were positive. After false discovery rate correction, significant increases remained for position C in both frequency (2.62 to 3.34; *p* = 0.005) and pleasure (3.10 to 3.80; *p* = 0.009), and for positions E (2.04 to 2.46; *p* = 0.010) and G (2.18 to 2.68; *p* = 0.006) in frequency only (Figure 2). These position-related findings should be interpreted as exploratory in nature.

The magnitude of weight loss was not significantly associated with the magnitude of improvement in erectile function or genital self-image. The Spearman correlation coefficients between ΔBMI or %TBWL and changes in IIEF-5 or MGSIS-7 ranged from −0.180 to 0.183, with all *p* values > 0.20. Likewise, changes in the IIEF-5, MGSIS-7, and HADS-M scores did not differ significantly according to baseline hypertension, type 2 diabetes mellitus, hyperlipidaemia, or cumulative comorbidity burden. In contrast, greater reductions in psychological distress were associated with greater improvements in erectile function (ΔHADS-M vs. ΔIIEF-5: ρ = −0.307, *p* = 0.030), whereas no significant association was observed between changes in HADS-M and the MGSIS-7.

Neither the magnitude of glycaemic improvement nor the degree of improvement in sleep-disordered breathing was associated with the change in any primary outcome. Change in glycated haemoglobin correlated with neither the change in IIEF-5 (ρ = −0.135, *p* = 0.350), MGSIS-7 (ρ = 0.102, *p* = 0.357), nor HADS-M (ρ = 0.267, *p* = 0.061), Participants in whom a metabolic comorbidity remitted showed a numerically larger improvement in erectile function than those in whom it did not (median change 4.5 versus 1.0 points; |r| = 0.359), although the difference was not significant and did not survive correction for multiplicity (*p* = 0.076, adjusted *p* = 0.531); no such signal was observed for genital self-image (*p* = 0.198) or psychological distress (*p* = 0.667) (Supplementary Table S1). In a multivariable model for change in the IIEF-5 adjusting for percentage total body weight loss, change in glycated haemoglobin, postoperative improvement in apnoea severity, follow-up duration, and age, the change in HADS-M score remained the only independent correlate (β = −0.467 per point, 95% CI [−0.707, −0.227], *p* < 0.001; adjusted R^2^ = 0.214, model *p* = 0.010; all variance inflation factors < 1.5). In the corresponding model for change in the MGSIS-7, percentage total body weight loss was the only nominally significant term (β = 0.129, *p* = 0.029), and the model did not reach significance overall (*p* = 0.218). Given six predictors at n = 50, these models are exploratory (Supplementary Table S2).

Sensitivity analyses confirmed the robustness of the primary findings. Replication of the analyses in the laparoscopic sleeve gastrectomy subgroup (n = 42) yielded results consistent in direction, magnitude, and statistical significance with those observed in the full cohort. Likewise, winsorisation of continuous change scores at the 5th and 95th percentiles resulted in only trivial changes in effect estimates and did not alter statistical significance. Overall, no substantive modification of any primary outcome was observed across the sensitivity analyses.

## 4. Discussion

This prospective cohort study demonstrated significant improvements in three key psychosexual domains: erectile function, genital self-image, and overall psychological distress. The most pronounced effect was observed on genital self-image, followed by psychological distress and erectile function. Exploratory sexual behaviour outcomes also suggested postoperative change, particularly through increased monthly coital frequency, although the increase in the proportion of sexually active men did not reach statistical significance, and position-related findings should be interpreted cautiously. Importantly, these benefits were not linearly related to the magnitude of weight loss and were not significantly associated with baseline cardiometabolic comorbidity, suggesting that postoperative sexual health gains may not be explained by kilograms lost alone but rather by a combination of metabolic recovery, improved body-related self-perception, and reduced psychological distress. The selective correlation between improvement in HADS-M and improvement in IIEF-5, but not MGSIS-7, suggests at least partially dissociable postoperative pathways, with erectile recovery appearing more closely related to changes in psychological distress, whereas changes in genital self-image may reflect broader shifts in body-related sexual self-perception.

Regarding erectile outcomes, our findings are directionally consistent with most bariatric studies using IIEF-based measures, although direct comparisons should be made cautiously because we used the abbreviated IIEF-5 rather than the 15-item version. In our cohort, the mean IIEF-5 score increased from 16.3 to 19.6, and the proportion of men without erectile dysfunction increased from 40.0% to 54.0%. These findings align with meta-analytic evidence showing postoperative improvements in IIEF-5 and IIEF-15 scores after bariatric surgery [4,13]. However, studies using the full IIEF-15 have indicated that postoperative recovery may be domain-specific, with orgasmic function often being less responsive than erectile function or satisfaction [4,17,18]. Thus, the present findings support the beneficial effect of bariatric surgery on erectile function while also highlighting the need for a broader psychosexual assessment beyond erectile function alone.

Importantly, the absence of a significant association between the magnitude of weight loss and improvement in psychosexual outcomes should not be interpreted as evidence that weight reduction is irrelevant. Rather, once clinically meaningful postoperative weight loss has been achieved, between-patient variability in sexual recovery may depend more on psychological, body image, hormonal, relational, and baseline sexual health factors than on kilograms lost alone. Prior literature also suggests that the predictor patterns are mixed. Gokalp et al. did not find a significant relationship between postoperative erectile function and age, preoperative BMI, smoking, alcohol use, comorbidity burden, or change in weight [6], whereas Efthymiou et al. found that improvement in total sexual satisfaction was not associated with baseline BMI or BMI reduction [16]. In contrast, Sarhan et al. reported that weight and BMI were significant predictors of IIEF scores [15]. Such heterogeneity likely reflects differences in study design, follow-up duration, sample size, procedure mix, and the use of the IIEF versus broader psychosexual or quality-of-life measures.

The magnitude of improvement in sleep-disordered breathing observed here is consistent with the established effect of bariatric surgery on obstructive sleep apnoea. Our normalisation rate of 33.3% is lower than the pooled remission estimate of approximately 65% reported in a recent meta-analysis [25], but the comparison is confounded by case mix and by endpoint definition. Nearly half of our affected participants had severe apnoea at baseline, a stratum in which normalisation to an index below 5 occurred in only 13.0%, whereas improvement by at least one grade was as frequent in this stratum as in the mildest one; the constituent studies of that meta-analysis, moreover, define remission variously as an index below 5, below 10, below 15, or as a halving from baseline. Judged on the endpoint of improvement rather than normalisation, our figure of 77.1% is fully concordant with the literature. Because obstructive sleep apnoea is itself associated with erectile dysfunction [26] and its treatment improves erectile function [27], we anticipated that men whose apnoea improved would show larger psychosexual gains. They did not. The polysomnographic basis of the grading makes measurement error an improbable explanation, yet the contrast available was narrow, since only 11 of 48 affected men failed to improve, and categorising a continuous index reduces statistical power; the analysis should therefore be read as exploratory rather than as evidence of no effect.

The use of genital self-image as a separate patient-reported outcome measure represents an important and distinctive aspect of the present study. To date, the MGSIS-7 has been used primarily in validation studies and in selected urological populations, whereas its application among men undergoing bariatric surgery remains limited. Sarwer et al. assessed body image and depressive symptoms in relation to IIEF scores, demonstrating substantial improvements, mainly during the first postoperative year after bariatric surgery, followed by the stabilisation of these effects [28]. Similarly, in a prospective study of men undergoing surgical treatment for phimosis, Czajkowski et al. reported improvements in both the IIEF-5 and MGSIS-7 scores after circumcision, supporting the clinical relevance of genital self-image in male sexual health research [21]. The present findings extend this construct to a bariatric population, in which genital self-image may be shaped not only by the perceived appearance of the genitalia but also by weight loss, changes in body habitus, improved mobility during sexual activity, increased self-confidence, and partner-related factors.

The analysis of sexual behaviours, including monthly coital frequency and predefined sexual positions, should be regarded as hypothesis-generating. Although the monthly coital frequency increased after surgery, the increase in the proportion of sexually active men did not reach statistical significance, and the position-related questionnaire was not independently validated. Therefore, these findings suggest a possible broadening of sexual activity after surgery rather than definitive evidence of changes in sexual repertoire or position-related satisfaction. Future studies should use validated instruments or formally developed patient-reported items to assess mobility, comfort, pleasure, and partner-related aspects of sexual activity after bariatric surgery.

This study had several notable strengths. This prospective study with repeated assessments of the same participants both prior to surgery and after a minimum of 12 months ensured complete paired data for all primary outcomes and no attrition. Erectile function, genital self-image, and psychological distress were evaluated using validated instruments, and the analysis extended beyond significance testing by reporting effect sizes with bias-corrected, bootstrap confidence intervals. Furthermore, multiplicity was addressed for the primary endpoints, enhancing the confidence that the observed postoperative improvements were not attributable to chance alone. However, several limitations must be acknowledged. This was a single-centre study lacking a non-surgical control group, and the majority of participants underwent sleeve gastrectomy, which constrains generalisability and procedure-specific interpretation. Outcomes were assessed at only two time points, precluding the determination of the time course and durability of postoperative psychosexual recovery and weight regain. The information regarding perioperative complications, reoperations, readmissions and nutritional supplementation has not been recorded. Neither sex hormones nor inflammatory markers were measured in this cohort. Total and free testosterone, sex hormone-binding globulin, luteinising hormone, follicle-stimulating hormone, and oestradiol were not part of the urological protocol, and no partial subset is available. Additionally, metabolic measures such as blood pressure, fasting glucose, fasting insulin, Homeostasis Model Assessment of Insulin Resistance (HOMA-IR) and the lipid panel were not part of the data collection protocol and are not available retrospectively. The data regarding medication use and continuous positive airway pressure were not recorded. Moreover, relationship satisfaction ere not assessed. Therefore, our findings should be interpreted with caution and not as evidence of a specific biological mechanism. Finally, all key outcomes were self-reported, no partner- or relationship-level data were available, and the use of the IIEF-5 rather than the full IIEF limits direct comparison with domain-based studies.

From a clinical standpoint, these findings indicate that postoperative bariatric care should encompass not only weight loss and metabolic outcomes but also sexual function, body-related sexual self-perception, and mental well-being. The observed correlation between reduced psychological distress and improved erectile outcomes underscores the need for a more integrated follow-up model in which psychosexual screening and psychological support are integral components of routine care. Future research should be multicentre, involve extended follow-up with repeated assessments, incorporate partner-reported outcomes, and combine patient-reported measures with hormonal, endothelial, and inflammatory biomarkers to elucidate the mechanisms and sustainability of postoperative sexual recovery.

## 5. Conclusions

In this single-centre prospective cohort of men undergoing bariatric surgery, erectile function, genital self-image, and psychological distress all improved at follow-up, alongside substantial reductions in body mass, glycated haemoglobin, obstructive sleep apnoea severity, and chronic medication burden. The psychosexual improvements were not associated with the magnitude of weight loss, the degree of glycaemic improvement, or the extent of improvement in sleep-disordered breathing, whereas a greater reduction in psychological distress was associated with greater improvement in erectile function and remained the only covariate associated with change in IIEF-5 in an exploratory multivariable model 5. Because this was an uncontrolled before-and-after study without a non-surgical comparator and without hormonal or inflammatory measurements, these results demonstrate an association between bariatric surgery and improved patient-reported psychosexual outcomes rather than establishing that the surgery or the weight loss itself caused those improvements. They support the integration of sexual health and mental health assessment into postoperative bariatric care and require confirmation in larger controlled cohorts with repeated follow-up, partner-reported outcomes, and biological markers.

## Figures and Tables

**Table 1:** Baseline Demographic, Anthropometric, Psychometric, and Clinical Characteristics of the Study Cohort (N = 50), Stratified by Surgical Approach (Laparoscopic Sleeve Gastrectomy versus Pooled Bypass Procedures).

Variable	Overall (N = 50)	LSG (n = 42)	Bypass Procedures (n = 8)
Age (years), M (SD)	44.2 (9.5)	43.6 (9.8)	47.4 (7.9)
Body mass (kg), M (SD)	138.8 (21.4)	140.1 (20.9)	132.1 (24.3)
Height (m), M (SD)	1.8 (0.1)	1.8 (0.1)	1.8 (0.1)
BMI (kg/m^2^), M (SD)	42.8 (7.6)	43.2 (7.9)	40.7 (5.8)
IIEF-5 total, M (SD)	16.3 (8.8)	16.9 (8.7)	13.5 (9.7)
MGSIS-7 total, M (SD)	19.1 (4.1)	19.5 (4.0)	16.9 (4.0)
HADS-M total, M (SD)	13.3 (6.8)	13.4 (7.1)	13.1 (5.7)
HADS Anxiety, M (SD)	6.0 (3.1)	5.9 (3.2)	6.5 (2.5)
HADS Depression, M (SD)	4.4 (3.4)	4.6 (3.5)	3.6 (2.3)
Relationship status, n (%)	–	–	–
Single	6 (12.0)	5 (11.9)	1 (12.5)
In a relationship	44 (88.0)	37 (88.1)	7 (87.5)
Educational attainment, n (%)	–	–	–
Primary	3 (6.0)	1 (2.4)	2 (25.0)
Lower secondary	1 (2.0)	1 (2.4)	0 (0.0)
Secondary	23 (46.0)	20 (47.6)	3 (37.5)
Vocational	7 (14.0)	5 (11.9)	2 (25.0)
Higher	16 (32.0)	15 (35.7)	1 (12.5)
Sexual orientation, n (%)	–	–	–
Heterosexual	49 (98.0)	42 (100.0)	7 (87.5)
Homosexual	1 (2.0)	0 (0.0)	1 (12.5)
Sexually active, n (%)	–	–	–
Yes	37 (74.0)	32 (76.2)	5 (62.5)
No	13 (26.0)	10 (23.8)	3 (37.5)
Obstructive sleep apnoea, n (%)	–	–	–
Yes	48 (96.0)	40 (95.2)	8 (100.0)
No	2 (4.0)	2 (4.8)	0 (0.0)
Hypertension, n (%)	35 (70.0)	–	–
Type II diabetes, n (%)	16 (32.0)	–	–
Hyperlipidaemia, n (%)	14 (28.0)	–	–

Continuous variables are presented as mean (SD), and categorical variables are presented as n (%). The Bypass procedures column denotes a pooled subgroup (n = 8) comprising one-anastomosis gastric bypass (OAGB; n = 6), laparoscopic Roux-en-Y gastric bypass (LRYGB; n = 1), and single-anastomosis sleeve ileal bypass (pSASI-S; n = 1); pooling was undertaken to permit meaningful between-group comparisons, given the small individual subgroup sizes. Hypertension, type II diabetes mellitus, and hyperlipidaemia were reported at the cohort level only, as they were derived from free-text clinical records via standardised pattern matching. Abbreviations: LSG, laparoscopic sleeve gastrectomy; OAGB, one-anastomosis gastric bypass; LRYGB, laparoscopic Roux-en-Y gastric bypass; pSASI-S, single-anastomosis sleeve ileal bypass; BMI, body mass index; IIEF-5, International Index of Erectile Function-5 (range 5–25; higher = better erectile function); MGSIS-7, Male Genital Self-Image Scale (range 7–28; higher = more favourable genital self-image); HADS-M, Hospital Anxiety and Depression Scale—Modified (range 0–48; lower = less psychological distress).

**Table 2:** Paired Pre- versus Postoperative Comparison of Clinical, Metabolic, and Sleep-Related Variables (N = 50).

Variable	n	Baseline (T0)	Follow-Up (T1)	Change	Statistic	*p*	*p* (FDR)	r	95% CI
Body mass (kg)	50	138.8 ± 21.4	102.7 ± 19.7	−36.1 ± 15.2	V = 0	<0.001	<0.001	−1.000	Not estimable
Body mass index (kg/m^2^)	50	42.8 ± 7.6	31.7 ± 6.8	−11.1 ± 4.8	V = 0	<0.001	<0.001	−1.000	Not estimable
Glycated haemoglobin (%)	50	5.89 ± 1.03	5.31 ± 0.43	−0.57 ± 0.78	V = 80	<0.001	<0.001	−0.864	[−0.958, −0.659]
Glycated haemoglobin, dysglycaemia subgroup (%)	16	6.58 ± 1.27	5.46 ± 0.48	−1.11 ± 1.00	V = 0	<0.001	<0.001	−1.000	Not estimable
HbA1c ≥ 6.5%, n (%)	50	11 (22.0%)	0 (0.0%)	−22.0 pp	χ^2^ = 9.09	0.003	0.003	–	–
OSA severity grade (0–3)	50	2.26 ± 0.83	1.18 ± 1.08	−1.08 ± 0.90	V = 12	<0.001	<0.001	−0.968	[−1.000, −0.827]
Obstructive sleep apnoea present, n (%)	50	48 (96.0%)	32 (64.0%)	−32.0 pp	χ^2^ = 14.06	<0.001	<0.001	–	–
Chronic medications, count	50	3.88 ± 3.32	2.10 ± 2.30	−1.78 ± 2.78	V = 9	<0.001	<0.001	−0.945	[−1.000, −0.769]

Continuous variables are presented as mean ± SD and were compared with the Wilcoxon signed-rank test; effect sizes are rank-biserial correlations with bias-corrected and accelerated bootstrap 95% confidence intervals (B = 10,000). Dichotomous variables were compared with McNemar’s test and are presented as n (%); pp = percentage points. Where every within-participant difference shared the same sign, r attains ±1 and the bootstrap interval is not estimable. OSA severity was graded ordinally (0 = none, 1 = mild, 2 = moderate, 3 = severe). *p* (FDR) is the Benjamini–Hochberg-adjusted *p* value within this family of eight comparisons. The dysglycaemia subgroup comprises participants with type 2 diabetes mellitus or documented impaired fasting glucose at baseline. Abbreviations: HbA1c, glycated haemoglobin; OSA, obstructive sleep apnoea; SD, standard deviation.

**Table 3:** MGSIS-7 Item-Level Pre–Post Comparisons.

Item	T0 Mdn (IQR)	T1 Mdn (IQR)	V	*p*
1. Positive self-assessment	3 (2–3)	3 (3–4)	210	<0.001
2. Appearance satisfaction	3 (2–3)	3 (3–4)	243	<0.001
3. Comfort with partner viewing	3 (3–3)	3 (3–4)	328	0.002
4. Size satisfaction	3 (2–3)	3 (3–4)	243	<0.001
5. Functional satisfaction	3 (2–3)	3 (3–4)	242	<0.001
6. Comfort with medical examination	2 (2–3)	3 (2–3)	221.5	<0.001
7. Absence of genital shame	3 (2–3)	3 (3–4)	180	0.002

All seven items demonstrated statistically significant postoperative improvements (*p* < 0.01). Items were scored on a 4 (1 = strongly disagree to strongly agree, total score is 28). Satisfaction cutoff for total score: ≥23. Mdn = median; IQR = interquartile range; V = Wilcoxon signed-rank statistic. MGSIS-7: Male Genital Self-Image Scale.

**Table 4:** Pre–Post Comparison of Primary Endpoint Scores of IIEF-5, MGSIS-7, and HADS-M.

Variable (Mean)	Before Surgery	After Surgery	*p*	r	95% CI
IIEF-5	16.3 (±8.8)	19.6 (±6.8)	<0.001	0.729	[0.392, 0.911]
MGSIS-7	19.1 (±4.1)	21.9 (±4.1)	<0.001	0.880	[0.680, 0.965]
HADS-M	13.3 (±6.8)	8.7 (±6.8)	<0.001	−0.767	[−0.929, −0.447]
Anxiety	6.0 (±3.1)	4.1 (±3.1)	<0.001	−0.685	[−0.876, −0.350]
Depression	4.4 (±3.4)	2.2 (±3.0)	<0.001	−0.761	[−0.932, −0.380]
Hostility	2.9 (±1.4)	2.0 (±1.6)	0.047	−0.390	[−0.704, 0.018]

Wilcoxon Signed-Rank Tests with Effect Sizes (N = 50); r = rank-biserial correlation with bias-corrected accelerated bootstrap 95% CI (B = 10,000); Abbreviations: IIEF-5 = International Index of Erectile Function-5 (range 5–25; higher = better; non-sexually active <5), MGSIS-7 = Male Genital Self-Image Scale (range 7–28; higher = better), HADS-M = Hospital Anxiety and Depression Scale–Modified (range 0–48; lower = better).

## Data Availability

Data available on request due to restrictions (e.g., privacy, legal or ethical reasons).

## Supplementary Materials

The following supporting information can be downloaded at: https://www.mdpi.com/article/10.3390/jcm15166276/s1, Supplementary Table S1. Association of Clinical, Metabolic, and Sleep-Related Variables with Change in Psychosexual Outcomes (N = 50). Supplementary Table S2. Multivariable Linear Models for Change in Erectile Function and Genital Self-Image, Including Metabolic and Sleep-Related Covariates (N = 50). STROBE Statement.

## Abbreviations

The following abbreviations are used in this manuscript:

Abbreviation	Full Term
BMI	Body Mass Index
CI	Confidence Interval
CRP	C-Reactive Protein
FDR	False Discovery Rate
HADS-M	Hospital Anxiety and Depression Scale–Modified
IIEF-5	International Index of Erectile Function-5
IL-6	Interleukin-6
IQR	Interquartile Range
LRYGB	Laparoscopic Roux-en-Y Gastric Bypass
LSG	Laparoscopic Sleeve Gastrectomy
M	Mean
Mdn	Median
MGSIS-7	Male Genital Self-Image Scale-7
OAGB	One-Anastomosis Gastric Bypass
pSASI-S	Single-Anastomosis Sleeve Ileal Bypass
SD	Standard Deviation
STROBE	Strengthening the Reporting of Observational Studies in Epidemiology
T0	Preoperative Assessment
T1	Postoperative Follow-up Assessment
TBWL	Total Body Weight Loss
%TBWL	Percentage Total Body Weight Loss
