# Supplementary material for: Changes in Erectile Function, Genital Self-Image, and Psychological Distress in Men After Bariatric Surgery: A Single-Centre Prospective Cohort Study
Source: J Clin Med. 2026 Aug 13;15(16):6276. doi: 10.3390/jcm15166276 (PMC13513422; doi:10.3390/jcm15166276)
Supplement: Supplementary file 1 [file jcm-15-06276-s001.zip › jcm-4413214-supplementary.pdf]

STROBE Statement

	Item No	Recommendation
**Title and abstract**	1	(*a*) *Indicate the study's design with a commonly used term in the title or the abstract*<br>Title: Changes in Erectile Function, Genital Self-Image, and Psychological Distress in Men After Bariatric Surgery: A Single-Centre Prospective Cohort Study
		(*b*) *Provide in the abstract an informative and balanced summary of what was done and what was found*<br>Abstract: Background: Severe obesity is a chronic condition that adversely affects physical health and quality of life, including sexual function. However, its impact on multidimensional psychosexual outcomes remains unexplored. This study aimed to assess changes in erectile function, genital self-image, and psychological distress in men who underwent bariatric surgery.<br>Methods: This prospective cohort study involved 50 male participants undergoing primary bariatric surgery who were evaluated within 30 days prior to the surgical procedure and at a minimum of 12 months postoperatively. Standardised questionnaires were used, including the International Index of Erectile Function-5 (IIEF-5), Male Genital Self-Image Scale (MGSIS-7), and Hospital Anxiety and Depression Scale–Modified (HADS-M). Secondary outcomes encompassed parameters of weight loss, sexual activity, and selected aspects of sexual behaviour, such as the utilisation of various sexual positions, their associated pleasure, and frequency.<br>Results: Significant postoperative improvements were observed in BMI reduction from 42.8 kg/m2 to 31.7 kg/m² ($P < 0.001$), erectile function (IIEF-5 improved from 16.3 to 19.6; $P < 0.001$), genital self-image (MGSIS-7 improved from 19.1 to 21.9; $P < 0.001$), and psychological distress (HADS-M reduction from 13.3 to 8.7; $P < 0.001$). Sexual activity increased from 37/50 (74.0%) to 43/50 (86.0%), although this change was not statistically significant ($P = 0.114$). After FDR correction, frequency increased for positions C, E, and G, whereas pleasure increased for position C. Weight loss magnitude was not significantly correlated with changes in the IIEF-5 or MGSIS-7 scores. A greater reduction in the HADS-M score correlated with a greater improvement in the IIEF-5 score ($\rho = -0.307$, $P = 0.030$).<br>Conclusions: Bariatric surgery is associated with improvements in erectile function, genital self-image, and psychological distress. These exploratory findings support the incorporation of psychosexual and mental health assessments into postoperative bariatric care.
**Introduction**		
Background/rationale	2	*Explain the scientific background and rationale for the investigation being reported*<br>Severe obesity is a chronic multisystem disease associated with cardiometabolic morbidity and impaired health-related quality of life, including sexual well-being. In men with obesity, sexual function is an important yet frequently under-recognised dimension of quality of life. Metabolic and bariatric surgery is now established as the most effective treatment for durable weight reduction in severe obesity, and its benefits appear to extend beyond body weight and remission of comorbidities to broader psychosocial and sexual health outcomes.<br>The relationship between obesity and male sexual dysfunction is complex, both biologically and psychosocially. Proposed mechanisms include androgen deficiency related to increased aromatase activity, endothelial dysfunction, chronic low-grade inflammation, insulin resistance, and obesity-related comorbidities, alongside poorer

		body image, lower self-esteem, anxiety, and depressive symptoms, all of which may adversely affect erectile function and sexual satisfaction.<br>Available studies using IIEF-based measures after bariatric surgery generally report postoperative improvement, although the effects vary across domains, procedures, and follow-up intervals. A 2023 systematic review and meta-analysis including 14 longitudinal studies and 508 patients demonstrated significant improvements in overall IIEF, erectile function, sexual desire, intercourse satisfaction, and overall satisfaction, whereas orgasmic function did not improve significantly. A more recent systematic review and meta-analysis including 21 studies and 695 participants likewise reported postoperative improvement in IIEF-5 and IIEF-15 scores and an increase in total testosterone levels, while emphasising heterogeneity in study design, surgical procedures, follow-up duration, and sample size. Individual prospective studies have similarly reported improvements after bariatric surgery, although postoperative recovery may be nonlinear and domain-specific.<br>Taken together, current evidence supports the beneficial effect of bariatric surgery on male sexual function; however, most previous studies have focused on IIEF outcomes, hormonal measures, health-related quality of life, or broader sexual satisfaction rather than on a multidimensional psychosexual construct. In particular, prospective studies simultaneously evaluating erectile function, genital self-image, and psychological distress in the same cohort of men remain scarce. It also remains unclear whether postoperative sexual improvement is explained primarily by the magnitude of weight loss or whether it is more closely related to changes in body-related sexual self-perception and psychological well-being.
Objectives	3	*State specific objectives, including any prespecified hypotheses*<br>The aim of this prospective cohort study was to assess longitudinal changes in erectile function, genital self-image, and psychological distress in men undergoing bariatric surgery, as measured by the IIEF-5, Male Genital Self-Image Scale-7 (MGSIS-7), and Hospital Anxiety and Depression Scale–Modified (HADS-M), respectively. The secondary aims were to evaluate monthly coital frequency and the use of different sexual positions and to explore whether postoperative changes in psychosexual outcomes were associated with the magnitude of weight loss, baseline cardiometabolic comorbidity, and changes in psychological distress. We hypothesised that bariatric surgery would be associated with improvements across all three psychosexual domains and that changes in erectile function would be more strongly associated with reductions in psychological distress than with the magnitude of weight loss.
**Methods**		
Study design	4	*Present key elements of study design early in the paper*<br>Single-centre prospective cohort study.
Setting	5	*Describe the setting, locations, and relevant dates, including periods of recruitment, exposure, follow-up, and data collection*<br>Study was conducted at the Medical University of Gdańsk (Department of General, Endocrine, and Transplant Surgery and Department of Urology). Men were recruited between 2023 and 2024 and reassessed at least 1 year after bariatric surgery. (…) This study enrolled 50 consecutive adult men who underwent primary bariatric surgery. The patients completed questionnaires 30 days before the surgery and at least 1 year after. The completion was self reported, without the influence of the researchers.
Participants	6	*Give the eligibility criteria, and the sources and methods of selection of participants. Describe methods of follow-up*<br>The inclusion criteria were age ≥18 years, eligibility for bariatric surgery, and written

		informed consent. The exclusion criteria were pharmacological treatment for erectile dysfunction, previously diagnosed and/or pharmacologically treated depression or anxiety, extensive reconstructive surgery in the genital area, and previous bariatric surgery.
Variables	7	*Clearly define all outcomes, exposures, predictors, potential confounders, and effect modifiers. Give diagnostic criteria, if applicable*<br>The primary outcomes focused on within-subject changes in erectile function, genital self-image, and psychological distress levels. These were assessed using the International Index of Erectile Function-5 (IIEF-5), where scores of 5-7 indicated severe erectile dysfunction, 8-11 moderate, 12-16 mild to moderate, 17-21 mild, and 22-25 no erectile dysfunction. Men who scored less than 5 points were not sexually active [13]. Genital self-image was assessed using the Male Genital Self-Image Scale (MGSIS-7), a seven-item instrument scored on a 4-point Likert scale, with total scores ranging from 7 to 28, with higher scores indicating more favourable genital self-image. A score of ≥23 was used to indicate satisfied genital self-image [14,15]. Psychological distress was assessed using the Hospital Anxiety and Depression Scale–Modified (HADS-M). Scores were interpreted as indicators of symptom burden rather than formal psychiatric diagnosis. For anxiety and depressive symptom subscales, scores of 0–7 were interpreted as normal, 8–10 as borderline, and ≥11 as elevated symptom burden, in line with the commonly used HADS interpretative thresholds. The HADS-M total score was analysed as a continuous measure of overall psychological distress [16,17]. Secondary outcomes included changes in body weight and body mass index (BMI), sexual activity status, monthly coital frequency, frequency and pleasure related to eight predefined sexual positions (Figure 2), and obstructive sleep apnoea status. Baseline hypertension, type 2 diabetes mellitus, and hyperlipidaemia were evaluated as potential effect modifiers. The percentage total body weight loss (%TBWL) was calculated as ((weight at T0 − weight at T1) / weight at T0) × 100.
Data sources/ measurement	8*	*For each variable of interest, give sources of data and details of methods of assessment (measurement). Describe comparability of assessment methods if there is more than one group*<br>The primary outcomes focused on within-subject changes in erectile function, genital self-image, and psychological distress levels. These were assessed using the International Index of Erectile Function-5 (IIEF-5), where scores of 5-7 indicated severe erectile dysfunction, 8-11 moderate, 12-16 mild to moderate, 17-21 mild, and 22-25 no erectile dysfunction. Men who scored less than 5 points were not sexually active. Genital self-image was assessed using the Male Genital Self-Image Scale (MGSIS-7), a seven-item instrument scored on a 4-point Likert scale, with total scores ranging from 7 to 28, with higher scores indicating more favourable genital self-image. A score of ≥23 was used to indicate satisfied genital self-image. Psychological distress was assessed using the Hospital Anxiety and Depression Scale–Modified (HADS-M). Scores were interpreted as indicators of symptom burden rather than formal psychiatric diagnosis. For anxiety and depressive symptom subscales, scores of 0–7 were interpreted as normal, 8–10 as borderline, and ≥11 as elevated symptom burden, in line with the commonly used HADS interpretative thresholds. The HADS-M total score was analysed as a continuous measure of overall psychological distress. Secondary outcomes included changes in body weight and body mass index (BMI), sexual activity status, monthly coital frequency, frequency and pleasure related to eight predefined sexual positions, and obstructive sleep apnoea status. Baseline

		hypertension, type 2 diabetes mellitus, and hyperlipidaemia were evaluated as potential effect modifiers. The percentage total body weight loss (%TBWL) was calculated as ((weight at T0 − weight at T1) / weight at T0) × 100.
Bias	9	*Describe any efforts to address potential sources of bias*<br>Procedure (bariatric surgery type) selection was based on clinical indications and surgeon judgement, which may have introduced selection bias.<br>(…)<br>However, several limitations must be acknowledged. This was a single-centre study lacking a non-surgical control group, and the majority of participants underwent sleeve gastrectomy, which constrains generalisability and procedure-specific interpretation. Outcomes were assessed at only two time points, precluding the determination of the time course and durability of postoperative psychosexual recovery. Additionally, hormonal or vascular-inflammatory biomarkers were not collected, although previous bariatric studies have included measures such as testosterone, estradiol, CRP, IL-6, and nitric oxide-related markers. Therefore, our findings should be interpreted with caution and not as evidence of a specific biological mechanism. Finally, all key outcomes were self-reported, no partner- or relationship-level data were available, and the use of the IIEF-5 rather than the full IIEF limits direct comparison with domain-based studies.
Study size	10	*Explain how the study size was arrived at*<br>No formal a priori sample size calculation was performed. The sample size was determined by the number of eligible men who underwent primary bariatric surgery during the recruitment period and completed paired follow-up. The observational cohort was not prospectively registered in a clinical trial
Quantitative variables	11	*Explain how quantitative variables were handled in the analyses. If applicable, describe which groupings were chosen and why*<br>Quantitative psychometric and anthropometric variables were analysed as continuous measures. Change scores (Δ = follow-up − baseline) were calculated for all primary outcomes and anthropometric indices. For descriptive analyses, IIEF-5 and HADS-M scores were additionally classified according to established clinical categories, whereas sexual activity was treated as a binary variable and measures of coital frequency and sexual-position use were analysed as ordinal variables.
Statistical methods	12	(*a*) *Describe all statistical methods, including those used to control for confounding*<br>Distributional assumptions were assessed using the Shapiro–Wilk test. Because the change scores were non-normally distributed and several outcomes were ordinal, the primary inferential framework was non-parametric. Pre–post comparisons were analysed using the Wilcoxon signed-rank test, with effect sizes expressed as rank-biserial correlations and 95% bias-corrected and accelerated bootstrap confidence intervals. Paired categorical variables were analysed using McNemar's exact test.
		(*b*) *Describe any methods used to examine subgroups and interactions*<br>Spearman's rank correlations were used to evaluate the associations between weight loss indices and changes in psychometric outcomes, as well as between changes in psychological distress and sexual outcomes. Change scores were compared between the comorbidity subgroups using the Mann–Whitney U test.
		(*c*) *Explain how missing data were addressed*<br>No missing data were observed for the primary outcomes. Complete paired data were available for all 50 participants. Therefore, no imputation procedures were required.
		(*d*) *If applicable, explain how loss to follow-up was addressed*<br>There was no lost to follow-up

		(e) *Describe any sensitivity analyses*<br>Sensitivity analyses were performed to evaluate the robustness of the findings. Primary outcome analyses were repeated in the laparoscopic sleeve gastrectomy subgroup, using parametric paired t-tests as an alternative analytical approach, and after winsorisation of continuous change scores at the 5th and 95th percentiles.
**Results**		
Participants	13*	(a) *Report numbers of individuals at each stage of study—eg numbers potentially eligible, examined for eligibility, confirmed eligible, included in the study, completing follow-up, and analysed*<br>This study enrolled 50 consecutive adult men who underwent primary bariatric surgery, and non of them was lost in the follow-up.
		(b) *Give reasons for non-participation at each stage*<br>All patients participated in each stage.
		(c) *Consider use of a flow diagram*<br>Flow chart is available.
Descriptive data	14*	(a) *Give characteristics of study participants (eg demographic, clinical, social) and information on exposures and potential confounders*<br>Table 1
		(b) *Indicate number of participants with missing data for each variable of interest*<br>No missing data were observed for the primary outcomes. Sexual position analyses were restricted to sexually active participants because non-sexually active participants did not complete these items.
		(c) *Summarise follow-up time (eg, average and total amount)*<br>The patients completed questionnaires 30 days before the surgery and at least 1 year after.
Outcome data	15*	*Report numbers of outcome events or summary measures over time*<br>Following surgery (at least one year after), mean body weight decreased from 138.8 ± 21.4 kg to 102.7 ± 19.7 kg, and mean BMI decreased from 42.8 ± 7.6 kg/m^2 to 31.7 ± 6.8 kg/m^2 (both $P < 0.001$).<br>(…)<br>The mean IIEF-5 score increased from 16.3 ± 8.8 at T0 to 19.6 ± 6.8 at T1, corresponding to a mean change of 3.2 ± 6.4 points (Wilcoxon $V = 640.5$, $P < 0.001$; rank-biserial $r = 0.729$, 95% CI [0.392, 0.911]).<br>(…)<br>Genital self-image also improved significantly, with the mean MGSIS-7 score increasing from 19.1 ± 4.1 to 21.9 ± 4.1 (mean change 2.8 ± 3.2; $V = 696.5$, $P < 0.001$; $r = 0.880$, 95% CI [0.680, 0.965]).<br>(…)<br>Psychological distress declined significantly, with the mean HADS-M decreasing from 13.3 ± 6.8 to 8.7 ± 6.8 (mean change −4.6 ± 7.0; $V = 115.5$, $P < 0.001$; $r = -0.767$, 95% CI [−0.929, −0.447]).<br>(…)<br>The proportion of sexually active men increased from 74.0% (n = 37) at baseline to 86.0% (n = 43) at follow-up. However, this change did not reach statistical significance in McNemar’s test ($P = 0.114$).<br>(…)<br>The monthly coital frequency increased significantly ($P = 0.016$; $r = 0.524$).<br>(…)<br>After false discovery rate correction, significant increases remained for position C in

		both frequency (2.62 to 3.34; P = 0.005) and pleasure (3.10 to 3.80; P = 0.009), and for positions E (2.04 to 2.46; P = 0.010) and G (2.18 to 2.68; P = 0.006) in frequency only.<br>(…)<br>The magnitude of weight loss was not significantly associated with the magnitude of improvement in erectile function or genital self-image. The Spearman correlation coefficients between ΔBMI or %TBWL and changes in IIEF-5 or MGSIS-7 ranged from −0.180 to 0.183, with all P values > 0.20. Likewise, changes in the IIEF-5, MGSIS-7, and HADS-M scores did not differ significantly according to baseline hypertension, type 2 diabetes mellitus, hyperlipidaemia, or cumulative comorbidity burden. In contrast, greater reductions in psychological distress were associated with greater improvements in erectile function (ΔHADS-M vs. ΔIIEF-5: ρ = −0.307, P = 0.030), whereas no significant association was observed between changes in HADS-M and the MGSIS-7.
Main results	16	(*a*) *Give unadjusted estimates and, if applicable, confounder-adjusted estimates and their precision (eg, 95% confidence interval). Make clear which confounders were adjusted for and why they were included*<br>See subpoint 15. No confounder-adjusted analyses were performed because the study used a within-subject pre–post design.
		(b) Report category boundaries when continuous variables were categorized<br>These were assessed using the International Index of Erectile Function-5 (IIEF-5), where scores of 5-7 indicated severe erectile dysfunction, 8-11 moderate, 12-16 mild to moderate, 17-21 mild, and 22-25 no erectile dysfunction. Men who scored less than 5 points were not sexually active [13]. Genital self-image was assessed using the Male Genital Self-Image Scale (MGSIS-7), a seven-item instrument scored on a 4-point Likert scale, with total scores ranging from 7 to 28, with higher scores indicating more favourable genital self-image. A score of ≥23 was used to indicate satisfied genital self-image [14,15]. Psychological distress was assessed using the Hospital Anxiety and Depression Scale–Modified (HADS-M). Scores were interpreted as indicators of symptom burden rather than formal psychiatric diagnosis. For anxiety and depressive symptom subscales, scores of 0–7 were interpreted as normal, 8–10 as borderline, and ≥11 as elevated symptom burden, in line with the commonly used HADS interpretative thresholds.
		(*c*) *If relevant, consider translating estimates of relative risk into absolute risk for a meaningful time period*<br>Not applicable. Relative risk estimates were not calculated.
Other analyses	17	*Report other analyses done—eg analyses of subgroups and interactions, and sensitivity analyses*<br>Sensitivity analyses confirmed the robustness of the primary findings. Replication of the analyses in the laparoscopic sleeve gastrectomy subgroup (n = 42) yielded results consistent in direction, magnitude, and statistical significance with those observed in the full cohort. Likewise, winsorisation of continuous change scores at the 5th and 95th percentiles resulted in only trivial changes in effect estimates and did not alter statistical significance. Overall, no substantive modification of any primary outcome was observed across the sensitivity analyses.
**Discussion**		
Key results	18	*Summarise key results with reference to study objectives*<br>This prospective cohort study demonstrated significant improvements in three key psychosexual domains: erectile function, genital self-image, and overall psychological

		distress. The most pronounced effect was observed on genital self-image, followed by psychological distress and erectile function. Exploratory sexual behaviour outcomes also suggested postoperative change, particularly through increased monthly coital frequency, although the increase in the proportion of sexually active men did not reach statistical significance, and position-related findings should be interpreted cautiously. Importantly, these benefits were not linearly related to the magnitude of weight loss and were not significantly associated with baseline cardiometabolic comorbidity, suggesting that postoperative sexual health gains may not be explained by kilograms lost alone but rather by a combination of metabolic recovery, improved body-related self-perception, and reduced psychological distress. The selective correlation between improvement in HADS-M and improvement in IIEF-5, but not MGSIS-7, suggests at least partially dissociable postoperative pathways, with erectile recovery appearing more closely related to changes in psychological distress, whereas changes in genital self-image may reflect broader shifts in body-related sexual self-perception.
Limitations	19	*Discuss limitations of the study, taking into account sources of potential bias or imprecision. Discuss both direction and magnitude of any potential bias*<br>This was a single-centre study lacking a non-surgical control group, and the majority of participants underwent sleeve gastrectomy, which constrains generalisability and procedure-specific interpretation. Outcomes were assessed at only two time points, precluding the determination of the time course and durability of postoperative psychosexual recovery. Additionally, hormonal or vascular-inflammatory biomarkers were not collected, although previous bariatric studies have included measures such as testosterone, estradiol, CRP, IL-6, and nitric oxide-related markers. Therefore, our findings should be interpreted with caution and not as evidence of a specific biological mechanism. Finally, all key outcomes were self-reported, no partner- or relationship-level data were available, and the use of the IIEF-5 rather than the full IIEF limits direct comparison with domain-based studies.
Interpretation	20	*Give a cautious overall interpretation of results considering objectives, limitations, multiplicity of analyses, results from similar studies, and other relevant evidence*<br>Regarding erectile outcomes, our findings are directionally consistent with most bariatric studies using IIEF-based measures, although direct comparisons should be made cautiously because we used the abbreviated IIEF-5 rather than the 15-item version. In our cohort, the mean IIEF-5 score increased from 16.3 to 19.6, and the proportion of men without erectile dysfunction increased from 40.0% to 54.0%. These findings align with meta-analytic evidence showing postoperative improvements in IIEF-5 and IIEF-15 scores after bariatric surgery. However, studies using the full IIEF-15 have indicated that postoperative recovery may be domain-specific, with orgasmic function often being less responsive than erectile function or satisfaction. Thus, the present findings support the beneficial effect of bariatric surgery on erectile function while also highlighting the need for a broader psychosexual assessment beyond erectile function alone.<br>Importantly, the absence of a significant association between the magnitude of weight loss and improvement in psychosexual outcomes should not be interpreted as evidence that weight reduction is irrelevant. Rather, once clinically meaningful postoperative weight loss has been achieved, between-patient variability in sexual recovery may depend more on psychological, body image, hormonal, relational, and baseline sexual health factors than on kilograms lost alone. Prior literature also suggests that the predictor patterns are mixed. Gokalp et al. did not find a significant relationship

		between postoperative erectile function and age, preoperative BMI, smoking, alcohol use, comorbidity burden, or change in weight, whereas Efthymiou et al. found that improvement in total sexual satisfaction was not associated with baseline BMI or BMI reduction. In contrast, Sarhan et al. reported that weight and BMI were significant predictors of IIEF scores. Such heterogeneity likely reflects differences in study design, follow-up duration, sample size, procedure mix, and the use of the IIEF versus broader psychosexual or quality-of-life measures.<br>The use of genital self-image as a separate patient-reported outcome measure represents an important and distinctive aspect of the present study. To date, the MGSIS-7 has been used primarily in validation studies and in selected urological populations, whereas its application among men undergoing bariatric surgery remains limited. Sarwer et al. assessed body image and depressive symptoms in relation to IIEF scores, demonstrating substantial improvements, mainly during the first postoperative year after bariatric surgery, followed by the stabilisation of these effects. Similarly, in a prospective study of men undergoing surgical treatment for phimosis, Czajkowski et al. reported improvements in both the IIEF-5 and MGSIS-7 scores after circumcision, supporting the clinical relevance of genital self-image in male sexual health research. The present findings extend this construct to a bariatric population, in which genital self-image may be shaped not only by the perceived appearance of the genitalia but also by weight loss, changes in body habitus, improved mobility during sexual activity, increased self-confidence, and partner-related factors.<br>The analysis of sexual behaviours, including monthly coital frequency and predefined sexual positions, should be regarded as hypothesis-generating. Although the monthly coital frequency increased after surgery, the increase in the proportion of sexually active men did not reach statistical significance, and the position-related questionnaire was not independently validated. Therefore, these findings suggest a possible broadening of sexual activity after surgery rather than definitive evidence of changes in sexual repertoire or position-related satisfaction. Future studies should use validated instruments or formally developed patient-reported items to assess mobility, comfort, pleasure, and partner-related aspects of sexual activity after bariatric surgery.
Generalisability	21	*Discuss the generalisability (external validity) of the study results*<br>From a clinical standpoint, these findings indicate that postoperative bariatric care should encompass not only weight loss and metabolic outcomes but also sexual function, body-related sexual self-perception, and mental well-being. The observed correlation between reduced psychological distress and improved erectile outcomes underscores the need for a more integrated follow-up model in which psychosexual screening and psychological support are integral components of routine care. Future research should be multicentre, involve extended follow-up with repeated assessments, incorporate partner-reported outcomes, and combine patient-reported measures with hormonal, endothelial, and inflammatory biomarkers to elucidate the mechanisms and sustainability of postoperative sexual recovery.
**Other information**		
Funding	22	*Give the source of funding and the role of the funders for the present study and, if applicable, for the original study on which the present article is based*<br>This research did not receive any specific grant from funding agencies in the public, commercial, or not-for-profit sectors.

*Give information separately for exposed and unexposed groups.

Note: An Explanation and Elaboration article discusses each checklist item and gives methodological background and published examples of transparent reporting. The STROBE checklist is best used in conjunction with this article (freely available on the Web sites of PLoS Medicine at http://www.plosmedicine.org/, Annals of Internal Medicine at http://www.annals.org/, and Epidemiology at http://www.epidem.com/). Information on the STROBE Initiative is available at http://www.strobe-statement.org.
