# Supplementary material for: Changes in Erectile Function, Genital Self-Image, and Psychological Distress in Men After Bariatric Surgery: A Single-Centre Prospective Cohort Study
Source: J Clin Med. 2026 Aug 13;15(16):6276. doi: 10.3390/jcm15166276 (PMC13513422; doi:10.3390/jcm15166276)
Supplement: Supplementary file 1 [file jcm-15-06276-s001.zip › Supplementary Tables S1 & S2.pdf]

Supplementary Table S1. Association of Clinical, Metabolic, and Sleep-Related Variables with Change in Psychosexual Outcomes (N = 50).

Panel A. Continuous and ordinal predictors (Spearman rank correlation)

Predictor	Outcome	ϱ	95% CI	P	P (FDR)
Total body weight loss (%)	Δ IIEF-5	0.183	[−0.107, 0.452]	.203	.745
Δ BMI (kg/m²)	Δ IIEF-5	−0.180	[−0.462, 0.107]	.211	.745
Baseline HbA1c (%)	Δ IIEF-5	−0.032	[−0.350, 0.293]	.824	.898
Δ HbA1c (%)	Δ IIEF-5	−0.135	[−0.412, 0.148]	.350	.745
Baseline OSA severity grade	Δ IIEF-5	−0.095	[−0.380, 0.200]	.513	.806
Δ OSA severity grade	Δ IIEF-5	−0.019	[−0.287, 0.246]	.898	.898
Δ number of chronic medications	Δ IIEF-5	−0.124	[−0.428, 0.174]	.391	.745
Follow-up duration (months)	Δ IIEF-5	−0.038	[−0.322, 0.252]	.794	.898
Age at surgery (years)	Δ IIEF-5	0.076	[−0.214, 0.365]	.599	.823
Baseline cardiometabolic burden (0–3)	Δ IIEF-5	0.120	[−0.162, 0.394]	.406	.745
Δ HADS-M total	Δ IIEF-5	−0.307	[−0.541, −0.029]	.030	.332
Total body weight loss (%)	Δ MGSIS-7	0.180	[−0.107, 0.441]	.211	.774
Δ BMI (kg/m²)	Δ MGSIS-7	−0.133	[−0.422, 0.173]	.357	.774
Baseline HbA1c (%)	Δ MGSIS-7	0.010	[−0.290, 0.318]	.945	.945
Δ HbA1c (%)	Δ MGSIS-7	0.102	[−0.207, 0.398]	.483	.774
Baseline OSA severity grade	Δ MGSIS-7	0.044	[−0.227, 0.313]	.760	.929
Δ OSA severity grade	Δ MGSIS-7	0.262	[−0.004, 0.511]	.067	.732
Δ number of chronic medications	Δ MGSIS-7	−0.113	[−0.388, 0.159]	.433	.774
Follow-up duration (months)	Δ MGSIS-7	−0.099	[−0.373, 0.190]	.493	.774
Age at surgery (years)	Δ MGSIS-7	0.017	[−0.239, 0.274]	.909	.945
Baseline cardiometabolic burden (0–3)	Δ MGSIS-7	−0.050	[−0.333, 0.243]	.733	.929
Δ HADS-M total	Δ MGSIS-7	−0.120	[−0.408, 0.168]	.405	.774
Total body weight loss (%)	Δ HADS-M	−0.137	[−0.413, 0.162]	.343	.571
Δ BMI (kg/m²)	Δ HADS-M	0.164	[−0.126, 0.438]	.256	.571
Baseline HbA1c (%)	Δ HADS-M	−0.210	[−0.473, 0.091]	.142	.475
Δ HbA1c (%)	Δ HADS-M	0.267	[−0.018, 0.513]	.061	.475
Baseline OSA severity grade	Δ HADS-M	−0.077	[−0.377, 0.225]	.593	.741
Δ OSA severity grade	Δ HADS-M	−0.211	[−0.455, 0.072]	.142	.475
Δ number of chronic medications	Δ HADS-M	0.001	[−0.266, 0.271]	.994	.994
Follow-up duration (months)	Δ HADS-M	−0.099	[−0.373, 0.167]	.493	.704
Age at surgery (years)	Δ HADS-M	0.023	[−0.250, 0.280]	.876	.973
Baseline cardiometabolic burden (0–3)	Δ HADS-M	0.138	[−0.125, 0.384]	.337	.571

Panel B. Dichotomous predictors (Mann–Whitney U test)

Predictor	Outcome	Group 0, Mdn	Group 1, Mdn	r	P	P (FDR)
Postoperative OSA improvement (≥ 1 grade)	Δ IIEF-5	3.0 (n = 11)	1.0 (n = 37)	0.044	.833	.957
Remission/improvement of metabolic comorbidity	Δ IIEF-5	1.0 (n = 13)	4.5 (n = 24)	−0.359	.076	.531
Baseline type 2 diabetes mellitus	Δ IIEF-5	1.0 (n = 34)	2.0 (n = 16)	−0.083	.644	.957
Baseline hypertension	Δ IIEF-5	1.0 (n = 15)	2.0 (n = 35)	−0.164	.364	.957
Baseline hyperlipidaemia	Δ IIEF-5	1.5 (n = 36)	1.0 (n = 14)	−0.012	.957	.957
Baseline HbA1c ≥ 6.5%	Δ IIEF-5	1.0 (n = 39)	4.0 (n = 11)	−0.093	.645	.957
Baseline relationship status (partnered vs single)	Δ IIEF-5	1.5 (n = 6)	1.0 (n = 44)	−0.023	.940	.957
Postoperative OSA improvement (≥ 1 grade)	Δ MGSIS-7	5.0 (n = 11)	2.0 (n = 37)	0.248	.215	.502
Remission/improvement of metabolic comorbidity	Δ MGSIS-7	1.0 (n = 13)	4.0 (n = 24)	−0.260	.198	.502
Baseline type 2 diabetes mellitus	Δ MGSIS-7	3.0 (n = 34)	2.0 (n = 16)	0.002	1.000	1.000
Baseline hypertension	Δ MGSIS-7	4.0 (n = 15)	3.0 (n = 35)	0.074	.684	.958
Baseline hyperlipidaemia	Δ MGSIS-7	3.0 (n = 36)	0.0 (n = 14)	0.089	.631	.958

Predictor	Outcome	Group 0, Mdn	Group 1, Mdn	r	P	P (FDR)
Baseline HbA1c ≥ 6.5%	Δ MGSIS-7	4.0 (n = 39)	0.0 (n = 11)	0.347	.080	.502
Baseline relationship status (partnered vs single)	Δ MGSIS-7	1.5 (n = 6)	3.0 (n = 44)	−0.034	.904	1.000
Postoperative OSA improvement (≥ 1 grade)	Δ HADS-M	−6.0 (n = 11)	−5.0 (n = 37)	−0.150	.461	.921
Remission/improvement of metabolic comorbidity	Δ HADS-M	−3.0 (n = 13)	−4.0 (n = 24)	0.090	.667	.933
Baseline type 2 diabetes mellitus	Δ HADS-M	−5.0 (n = 34)	−5.0 (n = 16)	−0.026	.892	.957
Baseline hypertension	Δ HADS-M	−6.0 (n = 15)	−3.0 (n = 35)	−0.347	.055	.383
Baseline hyperlipidaemia	Δ HADS-M	−5.0 (n = 36)	−5.0 (n = 14)	−0.012	.957	.957
Baseline HbA1c ≥ 6.5%	Δ HADS-M	−5.0 (n = 39)	−6.0 (n = 11)	0.128	.526	.921
Baseline relationship status (partnered vs single)	Δ HADS-M	0.0 (n = 6)	−5.0 (n = 44)	0.341	.183	.640

Panel A reports Spearman rank correlation coefficients with percentile bootstrap 95% confidence intervals (B = 5,000). Panel B reports median change scores by group with the rank-biserial correlation as the effect size. P (FDR) values are Benjamini–Hochberg-adjusted within each outcome family. Improvement in apnoea was evaluated among the 48 participants with baseline apnoea; remission of metabolic comorbidity among the 37 participants with hypertension, type 2 diabetes mellitus, or hyperlipidaemia at baseline. Group 0 and Group 1 denote the reference and index categories, respectively. All analyses are exploratory; the study was not powered for subgroup inference.

Supplementary Table S2. Multivariable Linear Models for Change in Erectile Function and Genital Self-Image, Including Metabolic and Sleep-Related Covariates (N = 50).

Model	Term	β	SE	95% CI	P	VIF
Δ IIEF-5	Intercept	12.120	18.833	[−25.860, 50.101]	.523	–
Δ IIEF-5	Total body weight loss (%)	0.078	0.104	[−0.131, 0.288]	.455	1.44
Δ IIEF-5	Δ HADS-M total	−0.467	0.119	[−0.707, −0.227]	< .001	1.07
Δ IIEF-5	Δ HbA1c (%)	−0.606	1.190	[−3.006, 1.793]	.613	1.30
Δ IIEF-5	Postoperative OSA improvement (yes vs no)	−1.227	1.894	[−5.047, 2.592]	.520	1.08
Δ IIEF-5	Follow-up duration (months)	−1.051	1.595	[−4.269, 2.166]	.513	1.29
Δ IIEF-5	Age at surgery (years)	0.013	0.099	[−0.186, 0.212]	.894	1.35
Δ MGSIS-7	Intercept	18.781	10.353	[−2.098, 39.660]	.077	–
Δ MGSIS-7	Total body weight loss (%)	0.129	0.057	[0.014, 0.244]	.029	1.44
Δ MGSIS-7	Δ HADS-M total	−0.043	0.065	[−0.175, 0.090]	.520	1.07
Δ MGSIS-7	Δ HbA1c (%)	−0.026	0.654	[−1.345, 1.294]	.969	1.30
Δ MGSIS-7	Postoperative OSA improvement (yes vs no)	−1.782	1.041	[−3.882, 0.317]	.094	1.08
Δ MGSIS-7	Follow-up duration (months)	−1.674	0.877	[−3.443, 0.095]	.063	1.29
Δ MGSIS-7	Age at surgery (years)	0.060	0.054	[−0.050, 0.169]	.277	1.35

Ordinary least squares regression with change scores as dependent variables. Δ IIEF-5 model: adjusted R^2 = 0.214, model F test P = .010, Shapiro–Wilk P for residuals = .097, non-constant variance test P = .730. Δ MGSIS-7 model: adjusted R^2 = 0.052, model F test P = .218, Shapiro–Wilk P = .062, non-constant variance test P = .693. Six predictors at n = 50 correspond to approximately eight observations per predictor; the models are exploratory and were not pre-specified as confirmatory. Variance inflation factors were below 1.5 for every term. Improvement in apnoea was coded 1 for a decrease of at least one severity grade and 0 otherwise. Abbreviations: CI, confidence interval; SE, standard error; VIF, variance inflation factor.
